# Clinical and contrast-enhanced image features in the prediction model for the detection of small hepatocellular carcinomas

**DOI:** 10.7150/jca.47245

**Published:** 2020-10-18

**Authors:** Ming-Feng Chiang, Tse-Kai Tseng, Chia-Wen Shih, Tzeng-Huey Yang, Szu-Yuan Wu

**Affiliations:** 1 Division of Gastroenterology and Hepatology, Department of Internal Medicine, Lo-Hsu Medical Foundation, Lotung Poh-Ai Hospital, Yilan, Taiwan.; 2 Department of Radiology, Lo-Hsu Medical Foundation, Lotung Poh-Ai Hospital, Lotung, Taiwan.; 3 Department of Pathology, Lo-Hsu Medical Foundation, Lotung Poh-Ai Hospital, Lotung, Taiwan.; 4 Department of Food Nutrition and Health Biotechnology, College of Medical and Health Science, Asia University, Taichung, Taiwan; 5 Division of Radiation Oncology, Lo-Hsu Medical Foundation, Lotung Poh-Ai Hospital, Yilan, Taiwan; 6 Big Data Center, Lo-Hsu Medical Foundation, Lotung Poh-Ai Hospital, Yilan, Taiwan; 7 Department of Healthcare Administration, College of Medical and Health Science, Asia University, Taichung, Taiwan; 8 Cancer Center, Lo-Hsu Medical Foundation, Lotung Poh-Ai Hospital, Yilan, Taiwan; 9 Graduate Institute of Business Administration, Fu Jen Catholic University, Taipei, Taiwan.

**Keywords:** small HCC, venous phase, washout appearance, arterial phase, enhancement

## Abstract

**Purpose**: To identify novel radiological features and clinical characteristics to improve diagnostic criteria for early detection of small hepatocellular carcinoma (HCC).

**Patients and Methods**: We retrospectively recruited asymptomatic patients with no history of HCC but a high risk of HCC in whom a new, solitary, well-defined, solid nodule between 10 and 20 mm was detected through a screening ultrasound. We retrospectively collected all clinical data, and patients were examined using dynamic contrast-enhanced computed tomography or magnetic resonance imaging; subsequently, fine-needle biopsy was performed. A multivariate analysis of the predictors of small HCCs was performed by fitting a multiple logistic regression model with the stepwise variable selection method.

**Results**: In total, 392 and 347 patients with a small liver nodule received a final pathologic confirmation of HCC and non-HCC, respectively. The estimated odds ratios and 95% confidence intervals of tumor size > 12.45 mm, age > 56.61 years, liver cirrhosis, hepatitis C virus (HCV) carrier status, ln alpha-fetoprotein (AFP) > 1.954, arterial phase enhancement, and portal or venous phase washout appearance without arterial phase enhancement were 2.0735 (1.4746-2.9155), 1.8878 (1.2949-2.7521), 1.6927 (1.1294-2.5369), 1.6186 (1.0347-2.5321), 2.0297 (1.3342-3.0876), 3.7451 (2.3845-5.8821), and 2.0327 (1.3500-3.0608), respectively. The area under the receiver operating characteristic curves for the diagnosis of small HCCs was 0.79 for arterial phase enhancement and 0.75 for portal or venous phase washout appearance without arterial phase enhancement.

**Conclusion**: Clinical and contrast-enhanced image features are valuable in the prediction model for the detection and early diagnosis of small HCCs in patients with a high risk of HCC. In addition to negative portal or venous washout and negative arterial enhancement in images, age > 56.61 years, tumor size > 12.45 mm, HCV carrier status, and ln(AFP) > 1.954, are useful indicators for the early detection of small HCCs.

## Introduction

Hepatocellular cancer (HCC) is the fifth and ninth most frequently diagnosed cancer globally in adult men and women, respectively 1. HCC is the fourth leading cause of cancer-related death worldwide 2. Regions with a high incidence of HCC (more than 15 cases per 100,000 people per year) include sub-Saharan Africa, the People's Republic of China, Hong Kong, and Taiwan 3. Per 100,000 people, the incidence is 24.2 in parts of Africa and 35.5 in eastern Asia 4.

In Taiwan, HCC is the second most prevalent cancer 5. A national surveillance program has been in operation for several decades, and HCC can be detected even when the tumor is small (diameter < 2 cm) 6. Various curative modalities are suitable for small HCC, including surgical resection and local ablation, which can yield overall 5-year survival rates of 50%-70% 7, 8. Nonsurgical curative local ablation therapies are also advocated for small HCCs that are unresectable because of comorbidities, the patient's desire to preserve liver function, or the patient's refusal to surgical treatment. Percutaneous ethanol injection and radiofrequency ablation (RFA) are two of the most common local ablation modalities 9, 10. For small HCCs, RFA yields survival rates equivalent to those of surgical resection 11-13. Therefore, RFA has been advocated as a first-line curative therapy for small HCC 9, 10. Identifying significant clinical predictors and specific radiological features for the early detection of small HCCs is thus crucial for early treatment to mitigate liver damage and preserve liver function 14.

The diagnosis of small HCC can be difficult and often requires multiple imaging modalities 15. Tumors should be detected when they are sized <2 cm to enable all treatment options. However, HCC is frequently diagnosed late because of the absence of symptoms and the reluctance of many primary care physicians to provide surveillance for their high-risk patients 16-19. Therefore, some patients present with incurable HCC at the time of diagnosis. Thus, early detection of small HCCs is crucial for local treatment in these patients. Early detection of small HCC increases the chance of treatment^20^ and further improves overall survival in patients with HCC 21.

The early detection of small HCCs remains difficult when using typical imaging criteria for HCC according to the Liver Imaging Reporting and Data System (LI-RADS) algorithm 22, 23. In our daily clinical practice, approximately half of pathologically confirmed small HCCs exhibit no arterial phase enhancement in contrast-enhanced (CE) images; for such small liver nodules, regular follow-up is recommended until tumor growth is observed according to the LI-RADS algorithm 24. According to LI-RADs guidelines 25, for lesions <2 cm, sensitivity of HCC diagnosis decreases to 47% 26. For the characterization of lesions detected in patients with chronic liver disease with other significant clinical features, the positive predictive value for HCC is 97% 27, 28. The sensitivity of HCC diagnosis differs for patients with indeterminate- or intermediate-risk lesions between 1 and 2 cm, reflecting the uncertainty concerning the optimal predictors in patients with lesions of this size. Thus, the aim of this study was to identify the predictive value of some radiological features and combine them with clinical characteristics to improve diagnostic criteria for the early detection of small HCCs in Taiwan.

## Patients and Methods

### Patients

Between January 2007 and December 2016, we retrospectively recruited 739 asymptomatic patients with a high risk of HCC who had liver cirrhosis, hepatitis B virus (HBV), or HCV with no history of HCC, and in whom a new, solitary, well-defined, solid nodule sized between 10 and 20 mm was detected through a screening ultrasound (US) 29. Patients with multiple liver nodules were excluded. In this study, HBV infection was defined as hepatitis B surface antigen positive, and HCV infection was defined as HCV RNA positive or anti-HCV negative. We defined liver cirrhosis by using US findings such as surface nodularity, overall coarse and heterogeneous echotexture segmental hypertrophy, and atrophy. In accordance with their understanding of US features, two gastroenterologists or radiologists reviewed all US findings to obtain a consensus regarding liver cirrhosis. Patients with contraindications to CE computed tomography (CT), magnetic resonance imaging (MRI), or fine-needle biopsy (FNB) were excluded. Our protocols were reviewed and approved by the Institutional Review Board of Cathay General Hospital (CGH-LP No. 106003). After the detection of liver nodules through a screening US, we retrospectively collected clinical data. Patients were examined using dynamic CE CT or CE MRI and subsequently underwent FNB. The biopsy result was considered the gold standard for diagnosis, and we repeated the analysis for inconclusive diagnoses. For nodules without pathological confirmation, 3 months of screening US and 6 months of CE CT or CE MRI follow-up were implemented, and a new FNB was performed only when growth was detected during the follow-up. In such cases, we considered CT or MR findings of the previous study before the diagnostic biopsy. A flowchart of our screening procedures and the relevant criteria is provided in Supplemental Figure 1.

### Image acquisition

Dynamic CE MRI was performed in all patients by using the 1.5-T MRI system (Espree, Siemens Medical Systems, Erlangen, Germany) with a phased-array coil for signal detection. All patients underwent transverse T1-weighted in-phase (repetition time/time to echo [TR/TE], 100/5.24; matrix, 256 134; flip angle, 70°) and opposed-phase gradient echo (TR/TE, 100/2.38; matrix, 256 134; flip angle, 70°) with a slice thickness of 5 mm; transverse T2-weighted breath-hold gradient echo (TR/TE, 1100/116 or 1010/191; matrix, 256 144) with a slice thickness of 5 mm; and axial dynamic multiphasic CE three-dimensional (3D) T1 gradient echo of the liver with fat suppression in arterial, portal, and delayed phases (volumetric interpolated breath-hold examination sequence in cases of symphony and 3D breath-hold fast spoiled gradient echo in cases of SIGNA) with a slice thickness of 2.5 mm. Gadolinium (gadodiamide, 0.5 mmol/L; Ominscan-Amersham, Madrid, Spain) was injected at a dose of 0.2 mL/kg and at a rate of 2 mL/s. Last, a T1-weighted two-dimensional gradient echo MRI with fat suppression was performed 5 min after contrast injection (TR/TE, 160/2.6; matrix, 256 115).

Dynamic CE CT was performed using a multidetector 256-slice CT system (Definition Flash, Siemens Medical Systems). Arterial, portal, and venous phase images were obtained using a helical scanning technique with a beam collimation width of 5 mm, a pitch of 1:1.4, and a continuous 5-mm reconstruction; delayed phase images were obtained using an incremental (cluster) scanning technique with a slice thickness of 5 mm and a gap of 2 mm. We injected 120 mL of iohexol (300 mg I/mL) at a rate of 4 mL/s, and arterial phase imaging was performed 30 s after the start of the contrast material injection. The delay time was 68-70 s for portal or venous phase imaging and 5 min for delayed phase imaging. The selection of either CE CT or MRI was based on the respiratory control of each patient or other contraindications for MRI, such as having a metallic foreign body implant, gastric reflux device, insulin pumps, and cardiac pacing leads. The subsequent CE images for surveillance of HCC in the patients were the same as the initial CE CT or MR images. Arterial enhancement was defined as hyperintense findings relative to the surrounding liver parenchyma on arterial phase. Washout appearance was defined as a visually assessed temporal reduction in enhancement relative to the surrounding liver from an earlier to a later phase, resulting in portal venous or delayed phase hypoenhancement. Arterial phase enhancement was referred to as nonrim arterial hyperenhancement (nonrim APHE), and portal or venous washout appearance was named nonperipheral washout appearance. Portal or venous washout appearance can be assessed in either portal venous or delayed phase by using CT or MRI with the administration of a contrast agent. However, when using a gadolinium-based contrast medium, washout can only be assessed in the portal venous phase and cannot be reliably evaluated in the transitional or hepatobiliary phases.

### Fine-needle biopsy

FNB was indicated for patients with LI-RADS ≥ 3, in accordance with the criteria outlined in Supplemental Figure 1. Expert gastroenterologists performed FNB by using a 20-gauge needle (Yale Spinal BD medical, NJ, USA). When location and accessibility allowed, a core biopsy was performed using an 18-gauge needle (Monopty; Bard Inc., Covington, UK). Specimens were routinely processed and stained with hematoxylin-eosin. HCC diagnosis was conducted according to the criteria of the International Working Party. Lesions were divided according to FNB results into two groups (HCC and non-HCC lesions), which included all hepatic lesions except for HCC, without reference to benign or malignant etiology.

### Statistical analysis

Statistical analysis was performed using R 3.5.1 software (R Foundation for Statistical Computing, Vienna, Austria). In statistical testing, a two-sided *P* value of ≤.05 was considered statistically significant. The distributional properties of continuous variables are presented as the mean ± standard deviation, and categorical variables are presented as the frequency and percentage. In univariate analysis, the unadjusted effects of potential risk factors, prognostic factors, and predictors on the binary outcome were examined using the Wilcoxon rank-sum test, chi-square test, and Fisher's exact test, as appropriate for the data type. Subsequently, multivariate analysis was conducted by fitting a logistic regression model to estimate the adjusted effects of risk factors, prognostic factors, and predictors on the binary outcome, namely suicide attempt.

Our study was designed to compare the optimal cutoff values for tumor size, age, and ln alpha-fetoprotein (AFP) by maximizing the sum of sensitivity and specificity. We plotted a receiver operating characteristic (ROC) curve for analyzing the accuracy of tumor size, age, and ln(AFP). The area under the curve (AUC) for the ROC curve (AUROC) of each group was also calculated. For all comparisons, *P* < .05 was considered statistically significant.

The goal of regression analysis was to identify parsimonious regression models that fit the observed data sufficiently for effect estimation and outcome prediction. To ensure quality, basic model-fitting techniques were used in our regression analyses for (1) variable selection, (2) goodness-of-fit (GOF) assessment, and (3) regression diagnostics and remedies. To select predictors, estimate parameters, and avoid overfitting, we adopted five methods to analyze the data: subset selection, forward selection (a stepwise selection method), ridge regression, lasso regression, and elastic net. The accuracy metrics for the aforementioned methods are summarized in Supplemental Table 1. Six accuracy metrics were used to compare the performance of different methods: root mean square error (RMSE), mean absolute error (MAE), mean absolute percentage error (MAPE), Pearson correlation, and the correlation of increment. The stepwise selection method had the lowest RMSE, MAE, and MAPE and the highest correlation and correlation of increment, which indicated that it was the optimal method for explaining the data. Therefore, the stepwise variable selection procedure (with iterations between the forward and backward steps) was applied to obtain the optimal candidate for the final logistic regression model. All significant and nonsignificant relevant covariates in the univariate analysis and some of their interaction terms (or moderators) were included in the variable list for selection. To be conservative, significance levels for entry and stay were set to 0.15 or higher. Then, with the aid of substantive knowledge, the optimal candidate for the final logistic regression model was manually identified by individually removing covariates with *P* > .05 until all regression coefficients differed significantly from 0. Because the statistical testing at each step of the stepwise variable selection procedure was conditioned on other covariates in the regression model, the multiple testing problem was not of concern. Any discrepancy between the results of univariate and multivariate analyses was likely due to the confounding effects of uncontrolled covariates in the univariate analysis or the masking effects of intermediate variables (or mediators) in the multivariate analysis. Sensitivity analysis was conducted for the multivariate analysis of the predictors of small HCCs (1-2 cm), which was based on a multiple logistic regression model with different CE image features (Table 3).

**Figure 1:**
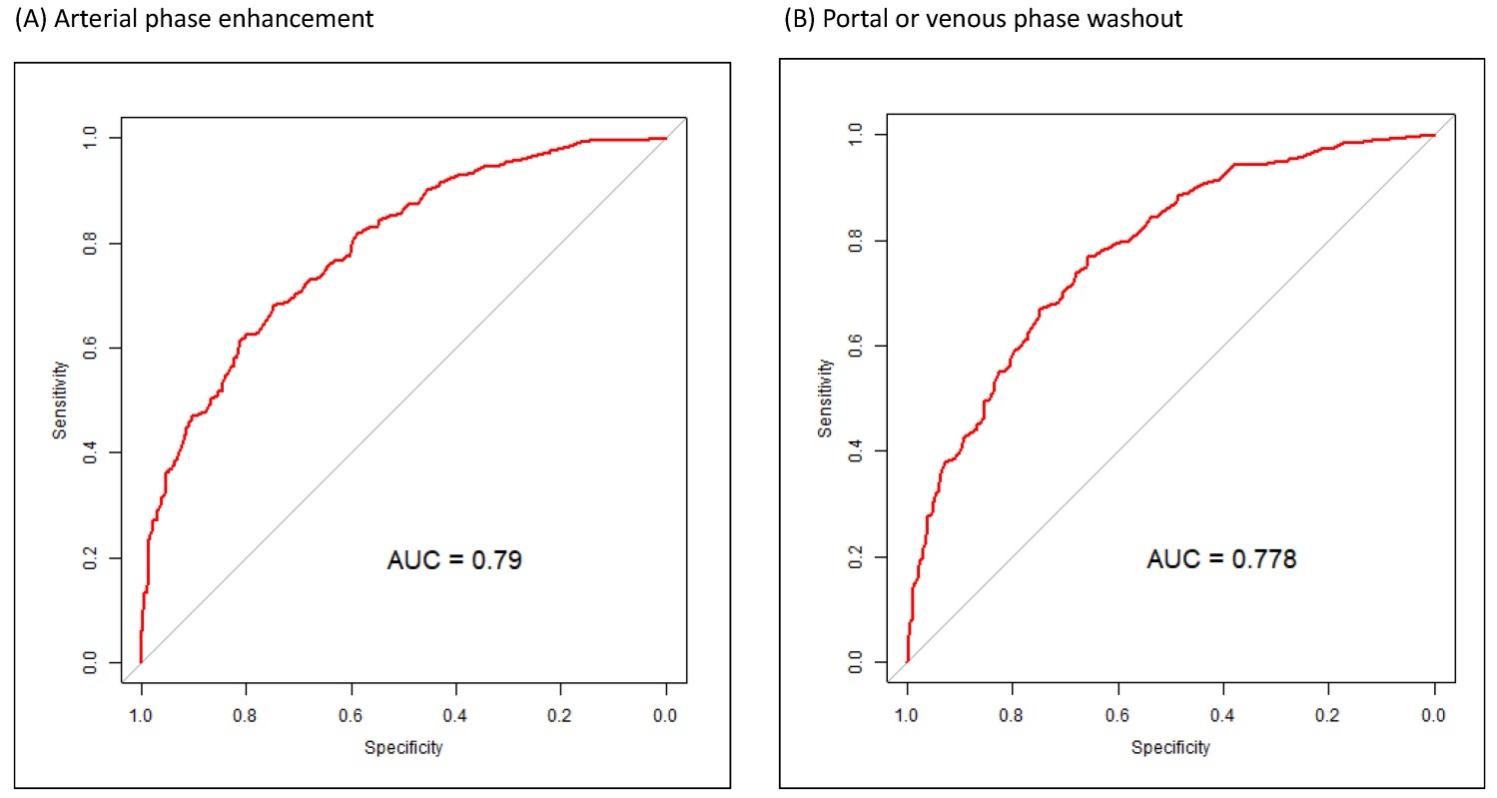

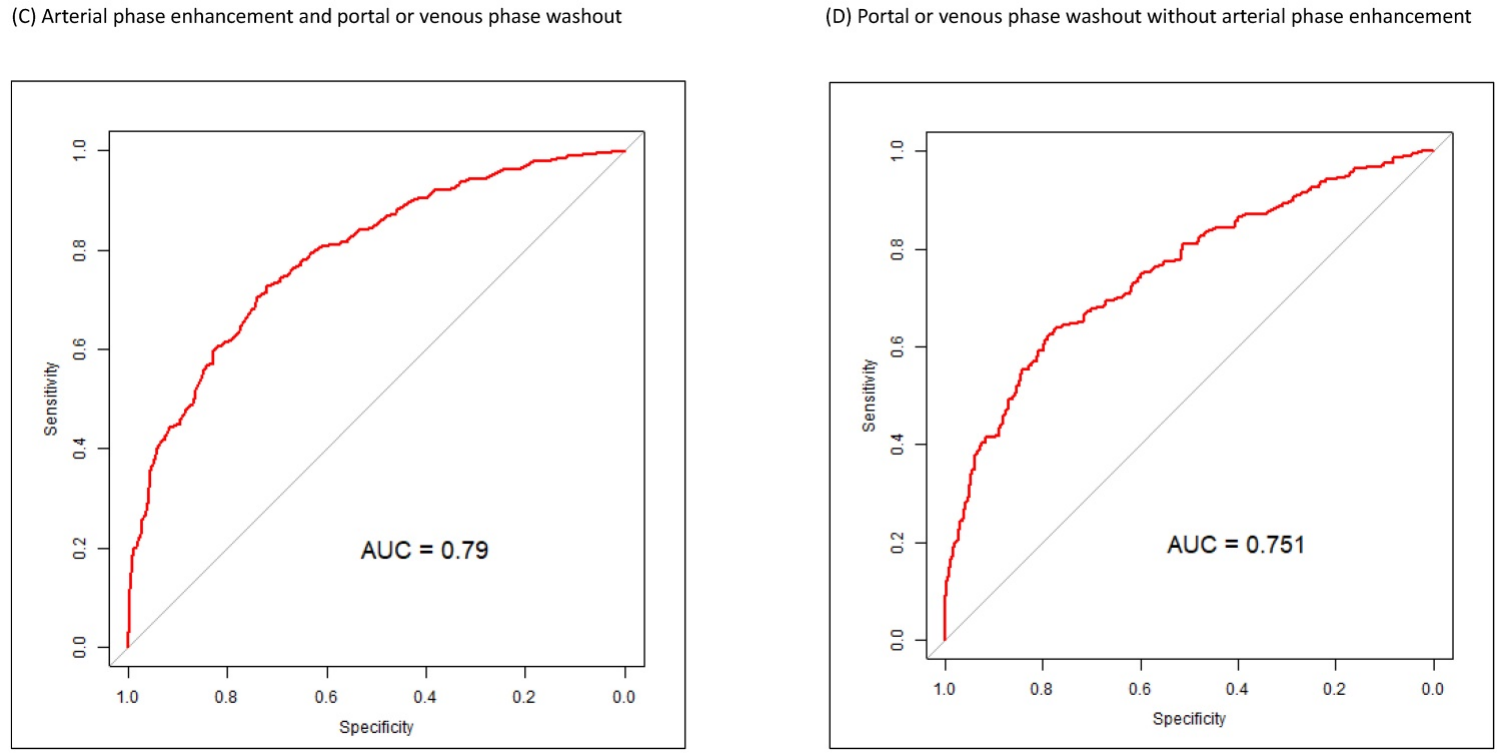
ROC curves for the diagnosis of small HCCs (<2 cm) in various arterial and portal or venous phases. (A) ROC curve for arterial phase enhancement in CT or MR images of small HCCs after pathologic proof was obtained. (B) ROC curve for portal or venous phase washout appearance in CT or MRI images of small HCCs after pathologic proof was obtained. (C) ROC curve for arterial phase enhancement and portal or venous phase washout appearance in CT or MR images of small HCCs after pathologic proof was obtained. (D) ROC curve for portal or venous phase washout appearance without arterial phase enhancement in CT or MR images of small HCC after pathologic proof was obtained. ROC, receiver operating characteristic; HCC, hepatocellular carcinoma; CT, computed tomography; MR, magnetic resonance.

During the stepwise variable selection procedure, simple and multiple generalized additive models (GAMs) were fitted to detect the nonlinear effects of continuous covariates and identify appropriate cutoff points for discretizing continuous covariates. The *vgam* function (Yee and Wild, 1996; Yee, 2014) was used to fit GAMs for our binary responses by using the VGAM package in R software with the default values of smoothing parameters 30-32. If a separation or high discrimination problem occurred in the logistic regression analysis, the exact logistic regression method was applied. Last, regression diagnostics for residual analysis, detection of influential cases, and a multicollinearity check were applied to identify model or data problems. Variance inflating factor (VIF) ≥ 10 for continuous covariates and VIF ≥ 2.5 for categorical covariates indicated a multicollinearity problem between covariates in the fitted logistic regression model. ROC curves were also used to determine the sensitivity and specificity for the diagnosis of small HCCs (<2 cm) in various arterial, portal, and venous phases (Figure 1).

## Results

A total of 739 patients with a mean solitary liver nodule size of 13.7 mm were recruited. In total, 221 and 518 patients underwent CE CT and CE MRI for surveillance of HCC, respectively. All 739 patients had LI-RADS ≥ 3 (Supplemental Figure 1) and received pathological confirmation. Of those recruited, 392 and 347 patients had small liver nodules that were eventually pathologically confirmed as HCCs and non-HCC liver nodules, respectively. Patients with small HCCs were older than patients with non-HCC liver nodules and had higher AFP levels, higher alanine transaminase (ALT) levels, higher aspartate aminotransferase (AST) levels, prolonged prothrombin time (PT), lower platelet counts, and larger tumor sizes (Table 1). Between 2014 and 2016, a greater proportion of patients were diagnosed as having small HCCs than as having non-HCC. Patients with small HCCs were more likely to be HCV carriers, have liver cirrhosis, and have Child-Pugh-Turcotte A-B than were those with non-HCC liver nodules. Patients with small HCCs had more CE CT or CE MR images exhibiting arterial phase enhancement, portal or venous phase washout appearance, arterial phase enhancement with portal or venous phase washout, and portal or venous phase washout appearance without arterial phase enhancement than did patients with non-HCC liver nodules (Table 1).

We adopted the following measures to assess the GOF of the fitted logistic regression model: (1) the estimated AUROC (also called the c statistic; 0 ≤ c ≤ 1), (2) the adjusted generalized R^2^ (0 ≤ R^2^ ≤ 1), and (3) the Hosmer-Lemeshow GOF test. Results with c ≥ 0.7 indicate an acceptable level of discrimination power. The values of adjusted generalized R^2^, as proposed by Nagelkerke (1991) 33, 34, are typically low for logistic regression models; however, an adjusted generalized R^2^ ≥ 0.30 indicates an acceptable fit for logistic regression models. In addition, higher p values in the Hosmer-Lemeshow GOF test indicate a better fit of the logistic regression model.

The multivariate analysis of the predictors of small HCCs (1-2 cm) was performed by fitting a multiple logistic regression model with the stepwise variable selection method. The results revealed several predictors of HCC diagnosis, namely tumor size > 12.45 mm, age > 56.61 years, liver cirrhosis, presence of HCV, ln(AFP) > 1.954, arterial phase enhancement in CE CT or CE MR images, and portal or venous phase washout appearance without arterial phase enhancement in CE CT or CE MR images (Table 2); their estimated odds ratios (ORs) and 95% confidence intervals (CIs) were 2.0735 (1.4746-2.9155), 1.8878 (1.2949-2.7521), 1.6927 (1.1294-2.5369), 1.6186 (1.0347-2.5321), 2.0297 (1.3342-3.0876), 3.7451 (2.3845-5.8821), and 2.0327 (1.3500-3.0608), respectively. Sensitivity analysis of the multivariate analysis indicated that arterial phase enhancement is a critical image predictor for detecting small (1-2 cm) HCC in high-risk patients regardless of portal or venous washout appearance, and portal or venous washout appearance is also a strong image predictor of small (1-2 cm) HCC regardless of arterial phase enhancement (Table 3). However, in the absence of arterial phase enhancement or portal or venous washout appearance, no significant prediction power for small (1-2 cm) HCC was observed.

Figure 1 presents the ROC curves for the diagnosis of small HCCs (1-2 cm) for arterial and portal or venous phases. The AUC for the diagnosis of small HCCs with pathologic evidence in CE CT or CE MR images was 0.79 for arterial phase enhancement, 0.78 for portal or venous phase washout appearance, 0.79 for arterial phase enhancement with portal or venous phase washout appearance, and 0.75 for portal or venous phase washout appearance without arterial phase enhancement (Figure 1).

## Discussion

For patients with a high risk of HCC and a screening US revealing a dominant, solid lesion with a size of ≥1 cm that has not been diagnosed as a hemangioma through CE imaging, practitioners typically obtain dynamic CE CT or CE MRI of the abdomen tailored for liver lesion evaluation 35. Biopsy for histologic confirmation is not required if the lesion fulfills the LI-RADS imaging criteria for HCC (LR-5) 36. However, if results indicate a possibility of malignancy (LR-M or LR-4) that might affect a patient's management, then a biopsy of the lesion may be indicated 36. Biopsy is not generally recommended for LR-1, LR-2, LR-3, and LR-5 lesions 36-39. For example, for patients with LR-3 lesions (38% likelihood of HCC), practitioners typically perform imaging with the same or an alternative modality every 3-6 months according to the LI-RADS algorithm 37, 40. For an LR-3 lesion, the American College of Radiology recommends repeated imaging until it can be downgraded on the basis of its stability or because of the evolution of imaging features to those of an LR-1 lesion (definitely benign) or an LR-2 lesion (probably benign); alternatively, the lesion's rating can be upgraded on the basis of the growth or evolution of imaging features to those of an LR-4, LR-5, or LR-M lesion 37, 40.

LR-4 lesions (74% likelihood of HCC) 37, 40 exhibit most but not all of the characteristic features of HCC. Lesions that are not arterially enhancing can also be categorized as LR-4 if they have a size of >2 cm and exhibit one of the three features or have a size of <2 cm and exhibit two of the three features 40. Therefore, arterial enhancement in CE CT or CE MRI is a valuable predictor for the diagnosis of small HCCs (<2 cm) according to the LI-RADS algorithm 40. The LI-RADS diagnostic criteria define small observations (<2 cm) with no APHE and exhibiting none or one of the three major features (enhancing capsule, nonperipheral washout, or threshold growth) as LR-3, whereas having all three features would result in an LR-4 categorization 40. Biopsy is not recommended for these small liver lesions with portal or venous phase washout appearance but without arterial enhancement 36-39. The results of the present study revealed a different perspective and highlighted the value of portal or venous phase washout appearance without arterial enhancement for detecting small HCCs in CE CT or CE MR images (Table 2 and Figure 1).

We found that arterial phase enhancement and venous or portal phase washout appearance in dynamic CT or MRI were independent predictors of the diagnosis of small HCCs (Table 2). In dynamic CE CT or CE MR images with only venous or portal phase washout appearance without arterial phase enhancement, the AUC of venous or portal phase washout appearance was very similar to that of arterial phase enhancement (Figure 1). Sensitivity analysis of the multivariate analysis indicated that arterial phase enhancement is a critical image predictor for detecting small (1-2 cm) HCC in high-risk patients regardless of portal or venous washout appearance, and portal or venous washout appearance is also a strong image predictor of small (1-2 cm) HCC regardless of arterial phase enhancement (Table 3). In our high-risk patients, portal or venous washout appearance was an independent predictor of small (1-2 cm) HCC. These findings, with potentially valuable implications for clinical practice, demonstrate the importance of portal or venous phase washout appearance in dynamic CE CT or CE MR images for small HCC in high-risk patients. The diagnostic sensitivity and specificity of portal or venous phase washout appearance were not inferior to those of arterial phase enhancement (Figure 1). Therefore, biopsy of small lesions with portal or venous washout appearance, even without arterial phase enhancement, is warranted (Tables 2 and 3 and Figure 1). According to our results, portal or venous washout appearance might be as critical as arterial phase enhancement in small liver lesions, and a biopsy of the lesion may be indicated. Early detection of HCC increases the chance of treatment^20^ and further improves overall survival in patients with HCC 21.

The characteristics of patients with small HCCs and those with non-HCC liver lesions differed in terms of age, AFP, ln(AFP), AST, ALT, PT (international normalized ratio), platelet count, and mean tumor size (Table 1). Diagnosis year, HCV carrier status, liver cirrhosis, and liver preserve advance also differed between patients with small HCCs and non-HCC liver lesions. The features of dynamic CE CT and CE MRI also differed between patients with small HCCs and non-HCC liver lesions, and some of these features, such as arterial phase enhancement, were compatible with the LI-RADS algorithm.^40^ The estimated ORs (95% CI) of tumor size (mm) > 12.45, age (years) > 56.608, liver cirrhosis (+), anti-HCV (+), and ln(AFP) > 1.954 were 2.0735 (1.4746-2.9155), 1.8878 (1.2949-2.7521), 1.6927 (1.1294-2.5369), 1.6186 (1.0347-2.5321, and 2.0297 (1.3342-3.0876), respectively. The ORs (95% CI) of CE image features of arterial phase enhancement and arterial phase without enhancement with portal or venous washout were 3.7451 (2.3845-5.8821) and 2.0327 (1.3500-3.0608), respectively (Table 2). Clinical features, such as higher AFP value and larger tumor size, observed in this study, are similar to those reported in previous studies for patients with a high risk of HCC alongside HBV, HCV, or liver cirrhosis requiring regular surveillance 15, 40, 41. HCC surveillance in high-risk individuals is commonly performed using the serum marker AFP and often in combination with images 15. Having hepatitis virus and liver cirrhosis is associated with an increased risk of HCC 41. The sex- and age-adjusted analysis of a Korean study indicated that cirrhosis increased the incidence of HCC 42-fold, HCV 19-fold, and each 5-year age increment 1.24-fold 42. Although older age has been identified as a risk factor for HCC in high-risk patients 42, 43. the present study is the first to demonstrate that age > 56.61 years is a clinical predictor of small HCCs in high-risk patients with HBV, HCV, or liver cirrhosis. In this study, tumor size > 12.45 mm, having HCV, and ln(AFP) > 1.954 were independent risk factors for small HCCs in high-risk patients (Table 2). Our ORs (Table 2) are smaller than those of other studies 15, 41-43, because our end-point was the detection of small HCC in high-risk patients, which is different from the risk of HCC that other studies employed. Independent risk factors for small HCCs in dynamic CT or MRI were arterial phase enhancement and portal or venous washout appearance regardless of arterial phase enhancement (Tables 2 and 3). Arterial phase enhancement is major indicator in the LI-RADS algorithm, and our outcomes are compatible with essential LI-RADS criteria 40. In our study, the sensitivity and specificity of portal or venous washout appearance without arterial phase enhancement were equal to those of arterial phase enhancement (Figure 1), which might be inconsistent with the LI-RADS algorithm.

The strength of this study is the identification of portal or venous phase washout appearance, regardless of arterial phase enhancement, as a new predictor for the early detection of small HCCs. Patients with clinical risk factors and image features (Table 2) should be encouraged to undergo FNB for further confirmation of small HCCs and to receive curative local treatment at an early stage. The sensitivity and specificity of portal or venous phase washout appearance without arterial phase enhancement were similar to those of the conventional image feature, arterial phase enhancement, for the diagnosis of small HCCs (Figure 1). Combining the two image features (Tables 2 and 3) with clinical risk factors (Table 2), such as age > 56.61 years, tumor size > 12.45 mm, having HCV, and ln(AFP) > 1.954, is practical and worthwhile for the early detection of small HCCs. Portal or venous phase washout without arterial phase enhancement is not included in the LI-4 category for 1-2-cm liver nodules. If patients with a high risk of HCC have portal or venous phase washout appearance without arterial phase enhancement, they should be considered as having an LI-3 lesion; therefore, FNB would not be indicated. The possibility of early small-HCC detection in these patients would thus be lower. Early treatment of small HCCs results in improved survival compared with the treatment of larger HCCs. The current study reveals the importance of portal or venous phase washout appearance without arterial phase enhancement in dynamic CE CT or CE MRI for the early diagnosis of small HCCs in patients with a high risk of HCC.

This study has several limitations. Because this was a retrospective review, our results might have been affected by selection bias. Associated limitations should be considered when interpreting our results. Two radiologists reviewed all dynamic CT or MR images to obtain a consensus regarding image features. In addition, patients with a higher probability of HCC might be more willing to receive an image exam and a liver biopsy. Therefore, our study might have recruited more patients with high levels of self-care (Supplemental Figure 1). However, the literature has no evidence that predictors of clinical and image features for HCC diagnosis may be associated with good or poor self-care. A further limitation of this study is the lack of detailed information regarding the overall survival of patients with small HCCs receiving early local treatment. Although the early detection of small HCCs could result in improved overall survival, the details of this effect remain unclear. Last, this was not a population-based study, and we focused on patients with a high risk of small liver nodules who were regularly followed up using dynamic CE CT or CE MRI. Despite these limitations, this study indicated that portal or venous phase washout appearance in dynamic CE CT or CE MRI has high sensitivity and specificity for the early diagnosis of small HCCs in patients with a high risk of HCC.

## Conclusions

Clinical and CE image features are valuable in the prediction model for the detection and early diagnosis of small HCCs (<2 cm) in patients with a high risk of HCC. MAPE age > 56.61 years, tumor size > 12.45 mm, HCV carrier status, and ln(AFP) > 1.954, are useful indicators for the early detection of small HCCs. In addition to positive portal or venous washout and positive arterial enhancement and negative portal or venous washout and positive arterial enhancement, negative portal or venous washout and negative arterial enhancement are suitable for the detection of early small HCC.

## Supplementary Material

Supplementary figure.

## Figures and Tables

**Table 1:** Demographic and clinical characteristics of patients with small HCCs (<2 cm) and non-HCC liver lesions.

Variable	All patients	HCC	Non-HCC	*P* value *
**Number of subjects (*n*)**	739 (100%)	392 (53.0%)	347 (47.0%)	
Age (years)	58.6 ± 12.9	61.2 ± 12.5	55.5 ± 12.6	<.0001
AFP (ng/mL)	253.31 (0.63-87580)	452.71 (0.98-87580)	28.05 (0.63-2950)	<.0001
ln (AFP)	2.12 ± 1.36	2.37 ± 1.45	1.83 ± 1.19	<.0001
AST	61.7 ± 57.1	65.5 ± 51.1	57.4 ± 63.0	.0005
ALT	54.1 ± 41.6	60.9 ± 46.5	46.5 ± 33.8	.0002
PT (INR)	1.103 ± 0.157	1.108 ± 0.160	1.099 ± 0.155	.0100
Platelet	152.4 ± 81.8	145.7 ± 78.3	160.0 ± 85.1	.0057
Tumor size (mm)/mean	13.7 ± 3.1	14.2 ± 3.1	13.0 ± 3.0	<.0001
**Sex**				.2894
Female	239 (32.3%)	134 (56.1%)	105 (43.9%)	
Male	500 (67.7%)	258 (51.6%)	242 (48.4%)	
**Diagnostic years**				<.0001
2007-2013	490 (66.3%)	218 (44.5%)	272 (55.5%)	
2014-2016	249 (33.7%)	174 (69.9%)	75 (30.1%)	
**Seasons**				.2449
Spring	218 (29.5%)	125 (57.3%)	93 (42.7%)	
Summer	214 (29.0%)	105 (49.1%)	109 (50.9%)	
Autumn	154 (20.8%)	86 (55.8%)	68 (44.2%)	
Winter	153 (20.7%)	76 (49.7%)	77 (50.3%)	
**Etiology**				
HBV	433 (58.6%)	215 (49.7%)	218 (50.3%)	.1338
HCV	236 (31.9%)	158 (66.9%)	78 (33.1%)	<.0001
**Alcohol**	253 (34.2%)	128 (50.6%)	125 (49.4%)	.3756
Other	5 (0.7%)	3 (60%)	2 (40%)	.7545
Unknown	12 (1.6%)	7 (58.3%)	5 (41.7%)	.7113
**Liver cirrhosis**				.0042
With	512 (69.3%)	290 (56.6%)	222 (43.4%)	
Without	227 (30.7%)	102 (44.9%)	125 (55.1%)	
**Advance of liver preserve**				.0162
Noncirrhotic	227 (30.7%)	102 (44.9%)	125 (55.1%)	
Child-Pugh-Turcotte class A	453 (61.2%)	257 (56.7%)	196 (43.3%)	
Child-Pugh-Turcotte class B	60 (8.1%)	33 (55.0%)	27 (45.0%)	
**Tumor location**				.4675
Segment 1	2 (0.27%)	1 (50.0%)	1 (50.0%)	
Segment 2	47 (6.4%)	30 (63.8%)	17 (36.2%)	
Segment 3	67 (9.1%)	39 (58.2%)	28 (41.8%)	
Segment 4	82 (11.1%)	45 (54.9%)	37 (45.1%)	
Segment 5	189 (25.6%)	97 (51.3%)	92 (48.7%)	
Segment 6	107 (14.5%)	62 (57.9%)	45 (42.1%)	
Segment 7	128 (17.3%)	62 (48.4%)	66 (51.6%)	
Segment 8	117 (15.8%)	56 (47.9%)	61 (52.1%)	
**Echogenicity**				.0718
Hypoechogenicity	497 (67.3%)	255 (51.3%)	242 (48.7%)	
Isoechogenicity	40 (5.4%)	17 (42.5%)	23 (57.5%)	
Hyperechogenicity	170 (23.0%)	98 (57.6%)	72 (42.4%)	
Mixed echogenicity	32 (4.3%)	22 (68.8%)	10 (31.3%)	
**Arterial phase enhancement**				<.0001
Positive	203 (72.5%)	140 (69.0%)	63 (31.0%)	
Negative	536 (27.5%)	252 (47.0%)	284 (53.0%)	
**Portal/venous washout appearance**				<.0001
Positive	311 (42.1%)	216 (69.5%)	95 (30.5%)	
Negative	428 (57.9%)	176 (41.1%)	252 (58.9%)	
**Arterial enhancement plus portal/venous washout**				<.0001
Yes	99 (13.4%)	88 (88.9%)	11 (11.1%)	
No	640 (86.6%)	304 (47.5%)	336 (52.5%)	
**Arterial phase no enhancement with portal/venous washout**				.0142
Yes	212 (28.7%)	128 (60.4%)	84 (39.6%)	
No	527 (71.3%)	264 (50.1%)	263 (49.9%)	
**Enhancing capsule appearance**				.0532
Yes	112 (15.2%)	67 (59.8%)	45 (40.2%)	
No	627 (84.8%)	286 (45.6%)	341 (54.4%)	

Data presented as mean ± standard deviation for continuous variables and frequency (percentage, %) for categorical variables. The *P* values of statistical tests were calculated using the Wilcoxon rank-sum test for continuous variables and Fisher's exact test for categorical variables. ln is the natural log function; ln(AFP) returns the power that *e* is raised by to obtain AFP. AFP, alpha-fetoprotein; ALT, alanine transaminase; AST, aspartate aminotransferase; PT, prothrombin time; INR, international normalized ratio; HBV, hepatitis B virus; HCV, hepatitis C virus; HCC, hepatocellular carcinoma; WD, well differentiated; MD, moderately differentiated; PD, poorly differentiated.

**Table 2:** Multivariate analysis for the predictors of small HCCs (<2 cm) based on a multiple logistic regression model with the stepwise variable selection method.

Covariate	*P* value	Estimated odds ratio	95% confidence interval of odds ratio
Intercept	<.0001	0.0900	**0.0530-0.1548**
**Clinical features**			
Tumor size (mm) > 12.45	<.0001	2.0735	**1.4746-2.9155**
Age (years) > 56.608	.0010	1.8878	**1.2949-2.7521**
Liver cirrhosis (+)	.0108	1.6927	**1.1294-2.5369**
Anti-HCV (+)	.0349	1.6186	**1.0347-2.5321**
ln(AFP) > 1.954	.0009	2.0297	**1.3342-3.0876**
**Contrast-enhanced image features**			
Arterial phase enhancement	<.0001	3.7451	**2.3845-5.8821**
Arterial phase no enhancement with portal/venous washout	<.0001	2.0327	**1.3500-3.0608**

ln is the natural log function; ln(AFP) returns the power that *e* is raised by to obtain AFP. AFP, alpha-fetoprotein; HCV, hepatitis C virus

**Table 3:** Sensitivity analysis of multivariate analysis for the predictors of small HCCs (1-2 cm) based on a multiple logistic regression model with different contrast-enhanced image features.

Contrast-enhanced image features	With arterial phase enhancement			Without arterial phase enhancement		
	*P* value	aOR^*^	95% CI of OR	*P* value	aOR^*^	95% CI of OR
With portal/venous washout appearance	<.0001	3.8160	1.4958-4.8876	<.0001	2.0327	1.3500-3.0608
Without portal/venous washout appearance	<.0001	2.1479	1.9791-4.9918	.7763	1.0306	0.6735-2.9642

*All covariates mentioned in Table 2 were adjusted

## Abbreviations

HCC: hepatocellular carcinoma
US: ultrasound
CE: contrast-enhanced
CT: computed tomography
MRI: magnetic resonance imaging
ORs: odds ratios
CI: confidence interval
HCV: hepatitis C virus
PEI: percutaneous ethanol injection
RFA: radiofrequency ablation
LI-RADS: Liver Imaging Reporting and Data System
HBV: hepatitis B virus
FNB: fine-needle biopsy
SD: standard deviation
GOF: goodness-of-fit
ROC: receiver operating characteristic
GAMs: generalized additive models
VIF: variance inflating factor
AFP: alpha-fetoprotein
ALT: alanine transaminase
AST: aspartate aminotransferase
PT: prolonged prothrombin time
AUC: area under the curve
RMSE: root mean square error
MAE: mean absolute error
MAPE: mean absolute percentage error
