# Supplementary figures and images for: Clinical and contrast-enhanced image features in the prediction model for the detection of small hepatocellular carcinomas
Source: J Cancer. 2020 Oct 18;11(24):7166–75. doi: 10.7150/jca.47245 (PMC7646160; doi:10.7150/jca.47245)

Supplemental Figure 1. Flowchart of screening patients with a high risk of HCC.

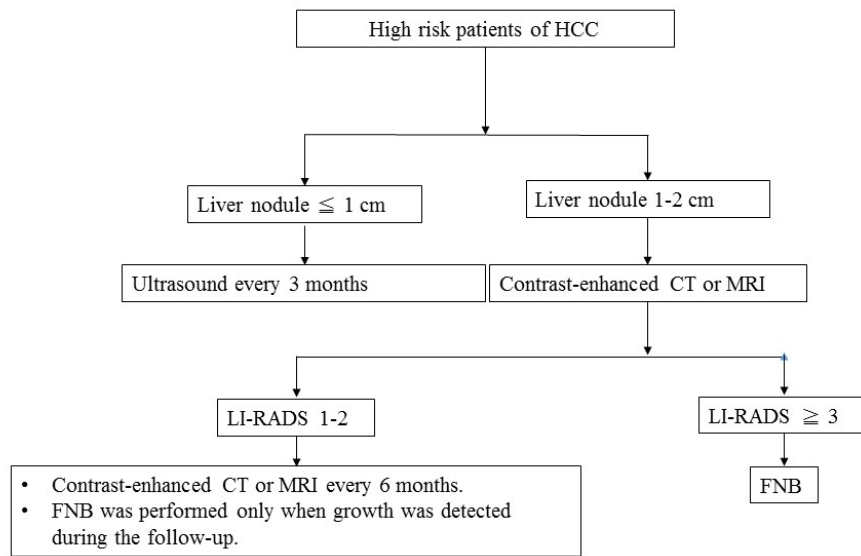

Supplement: Supplementary file 1 — Supplementary figure. [file jcav11p7166s1.pdf]
